# From Multi-Omics Approaches to Precision Medicine in Amyotrophic Lateral Sclerosis

**DOI:** 10.3389/fnins.2020.577755

**Published:** 2020-10-30

**Authors:** Giovanna Morello, Salvatore Salomone, Velia D’Agata, Francesca Luisa Conforti, Sebastiano Cavallaro

**Affiliations:** ^1^Institute for Research and Biomedical Innovation (IRIB), Italian National Research Council (CNR), Catania, Italy; ^2^Section of Pharmacology, Department of Biomedical and Biotechnological Sciences, University of Catania, Catania, Italy; ^3^Human Anatomy and Histology, University of Catania, Catania, Italy; ^4^Department of Pharmacy, Health and Nutritional Sciences, University of Calabria, Rende, Italy

**Keywords:** amyotrophic lateral sclerosis, ALS-FTD, personalized medicine, molecular taxonomy, multi-omics, systems biology

## Abstract

Amyotrophic lateral sclerosis (ALS) is a devastating and fatal neurodegenerative disorder, caused by the degeneration of upper and lower motor neurons for which there is no truly effective cure. The lack of successful treatments can be well explained by the complex and heterogeneous nature of ALS, with patients displaying widely distinct clinical features and progression patterns, and distinct molecular mechanisms underlying the phenotypic heterogeneity. Thus, stratifying ALS patients into consistent and clinically relevant subgroups can be of great value for the development of new precision diagnostics and targeted therapeutics for ALS patients. In the last years, the use and integration of high-throughput “omics” approaches have dramatically changed our thinking about ALS, improving our understanding of the complex molecular architecture of ALS, distinguishing distinct patient subtypes and providing a rational foundation for the discovery of biomarkers and new individualized treatments. In this review, we discuss the most significant contributions of omics technologies in unraveling the biological heterogeneity of ALS, highlighting how these approaches are revealing diagnostic, prognostic and therapeutic targets for future personalized interventions.

## Introduction

Amyotrophic lateral sclerosis (ALS) is a devastating and fatal neurodegenerative disease, characterized by the progressive deterioration of cortical and spinal motor neurons (MNs), leading invariably to progressive muscle weakness and paralysis. Death, often resulting from respiratory failure due to respiratory muscle weakness, generally occurs after 3–5 years from symptom onset, with only 5–10% of patients’ survival beyond 10 years (Brown and Al-Chalabi, 2017). ALS is the most common adult motor neuron disease with a worldwide annual incidence of about 2 per 100,000 persons and with an estimated prevalence of 5.4 per 100,000 individuals (Chiò et al., 2013). In most cases, mean age at onset is 50–60 years, while juvenile (before 25 years of age) and “young-onset” ALS cases (before 45 years), represent between∼1 and ∼10% of all patients, respectively (Artemiadis et al., 2016). No disease-modifying strategies are available so far, and therapies that can effectively stop or reverse the disease progression are urgently needed. The mainstay of treatment for ALS is mainly based on symptom management and respiratory support, with only two Food and Drug Administration (FDA)-approved treatments, riluzole, and edaravone, that appear to mildly slow disease progression and only in some patients (Bhandari et al., 2018; Dash et al., 2018; Jaiswal, 2019). The paucity of effective treatments has been attributed in part to the absence of complete knowledge of ALS pathogenesis, and in part to its heterogeneity with patients displaying widely distinct clinical features and progression patterns, together with a plurality of associated genes.

Over the last few years, the complexity of ALS has led to the concept of a spectrum of different disorders with different pathogenic mechanisms rather than a single disease. From a clinical point of view, in addition to typical or classic ALS (characterized by the simultaneous involvement of upper and lower motor neuron (UMN and LMN) at disease onset), several different phenotypic subtypes can be recognized based on the rate of progression, survival, age of onset, site of onset (bulbar vs. spinal) and prevalence of UMN or LMN motor signs (Brown and Al-Chalabi, 2017). Additionally, while ALS was historically judged as a pure motor neuron disease, it is now recognized that it represents a multi-systemic disorder affecting other brain regions, including frontotemporal, oculomotor, cerebellar, and/or sensory systems, and more rarely the basal ganglia and autonomic nervous system (Abrahams et al., 2014; Fang et al., 2017). To this regard, the most common alternative deficit observed in ALS patients is behavioral dysfunction and/or subtle cognitive impairment, which is also comorbid to ALS in about half of ALS individuals, and where a subset of ∼15% of patients receive the concomitant diagnosis of ALS with a frontotemporal dementia (FTD) syndrome (referred to as ALS-FTD or FTD-ALS patients) (Ferrari et al., 2011; Achi and Rudnicki, 2012; Chiò et al., 2019; Zucchi et al., 2019). The ALS-FTD relationship has been confirmed through genetic studies, suggesting these conditions can be viewed as divergent ends of the spectrum of a single clinically and etiologically heterogeneous condition (Ferrari et al., 2011).

Different clinical profiles are likely to reflect molecular heterogeneity in ALS. In fact, for example, the majority (∼90%) of ALS cases are sporadic (SALS), with unknown cause, while ∼10% of ALS patients show familiarity for the disease, usually transmitted according to an autosomal dominant inheritance (Ryan et al., 2018). However, this distinction is increasingly recognized to be artificial; FALS and SALS are, in fact, phenotypically indistinguishable and seem to show similar patterns of selective MN degeneration and vulnerability, and many mutations in one or more known FALS-associated genes have been found in SALS patients, suggesting the existence of common molecular mechanisms between these two disease forms (Renton et al., 2014; Kirby et al., 2016; Taylor et al., 2016). The complexity and heterogeneity of ALS also emerged from a pathophysiologic point of view, with a series of several biological and molecular pathways differently contributing to its development and progression. Despite the understanding of disease pathogenesis is far from exhaustive, numerous genetic and epidemiological risk factors have been identified, as well as various mechanisms have been suggested, including inflammatory and immune abnormalities, oxidative stress, mitochondrial dysfunction, glutamate excitotoxicity, proteasomal/autophagic impairment, defects in axonal transport and RNA metabolism (Taylor et al., 2016). With this in mind, it is clear that the current diagnostic classification criteria of ALS, primarily based on person’s signs and symptoms, are inadequate to characterize the complex and heterogeneous nature of ALS, as well as the use of a single compound to treat the patient population as a whole may hinder the identification of an effective therapy. Defining and stratification of ALS patients into disease subtypes cannot only provide important insights for diagnosis and prognosis but also for clinical trial planning and interpretation, thus achieving better care for ALS patients.

**FIGURE 1:**
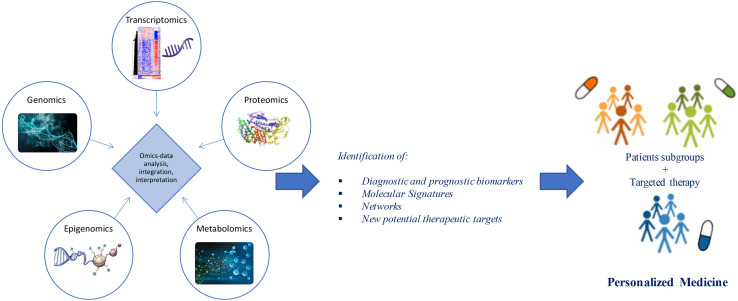
From omics technology to precision medicine in ALS. Multi-omics (e.g., genomics, transcriptomics, proteomics, epigenomics, metabolomics) data analysis and integration may allow patient stratification and targeted therapies. Through a “systems biology” approach, these technologies may move medicine from a “one-size-fits-all” toward a “personalized” model.

Advances in “omics” technologies (e.g., genome, transcriptome, proteome, epigenome, metabolome) and their correlation with the clinical phenotypes of the individual patient, are enabling medicine to move from a “one-size-fits-all” approach toward a “personalized” model, helping to clarify the molecular mechanisms underlying human disease and to provide both potential biomarkers and pharmacological targets for a more detailed patient stratification and personalized treatments (Figure 1). In this review, we discuss advances in the application of “-omics” to further our understanding of ALS, outline the evolving landscape of molecular classifications, and discuss how these techniques are contributing to reveal diagnostic and prognostic biomarkers and molecular targets for future personalized therapeutic interventions.

## Application of Omics: A Step Toward a Better Understanding of ALS Pathogenesis

Applications of omics platforms range from the detection of genes (genomics), mRNA (transcriptomics), proteins (proteomics), epigenomic factors (epigenomics), and metabolites (metabolomics). Thanks to omics technologies, it is now possible to quantify the amount of particular molecules (genes, mRNA, protein levels, and metabolites) of a biological system, and observe massive interactomes describing their complex interconnections. For complex and multifactorial pathologies such as ALS, the analysis and integration of different omics layers are crucial for the full knowledge of the disease, opening the way to the development of personalized diagnostic and therapeutic tools. Several omics studies have suggested multiple pathologic mechanisms associated to ALS, providing new insights into molecular signatures/markers and moving toward molecular-based classifications and tailored interventions.

### Genomics

The genomic landscape of ALS has been extensively surveyed, contributing to our understanding of ALS biological and clinical complexity. Analysis at this level requires not only the study of DNA sequence variations, including single nucleotide polymorphisms (SNPs) or mutations, but also genomic alterations and chromosomal changes, with consequent protein dysfunction or differences in concentration levels. Detailed information regarding ALS-related genes is available via the Amyotrophic Lateral Sclerosis Online Database (ALSOD)^1^. After the identification of mutations in the *SOD1* gene in 1993 (Rosen et al., 1993), more than 30 genes have been involved in the pathology, with the most common disease-causing variants in *C9orf72, SOD1, FUS, and TARDBP.* However, monogenic forms explain only a fraction of the diagnosed cases, suggesting ALS as a polygenic disease (McCann et al., 2017; Mejzini et al., 2019).

Thanks to the development of genome-wide association studies (GWAS) as well as the advances in massive parallel sequencing approaches, including whole-genome sequencing (WGS) and whole-exome sequencing (WES), enormous progress has been made in understanding genomics of ALS (Ramanan and Saykin, 2013; Cirulli et al., 2015; He et al., 2015; Butchbach, 2016; Van Rheenen et al., 2016; Little et al., 2017; Naruse et al., 2019; Bean et al., 2020). A growing number of causative and susceptibility genes have been identified so far in both familial and sporadic cases, the majority of which encode proteins implicated in cytoskeleton remodeling and axonal transport, mitochondrial metabolism and turnover, autophagy and proteostasis, membrane trafficking, RNA processing and DNA repair (Table 1; Ramanan and Saykin, 2013; Robberecht and Eykens, 2015; Maurel et al., 2018; Cook and Petrucelli, 2019; Mejzini et al., 2019; Gall et al., 2020). These genetic findings may guide patient stratification into different subgroups depending on which combination of pathways is deregulated, improving their recruitment for translational research and clinical trials (Vijayakumar et al., 2019; Volonté et al., 2020).

**TABLE 1:** Summary of the most known genes linked to ALS, their clinical phenotypes and affected pathway.

**Gene symbol**	**Gene name**	**Associated phenotype**	**Oxidative stress**	**Mito- chondria**	**Cytoskeleton and axonal dynamics**	**Protein trafficking and degradation**	**Autophagy**	**Vescicle trafficking**	**DNA repair**	**RNA processing**	**Innate immunity and neuro- inflammation**
SOD1	Superoxide dismutase 1	ALS, PMA, juvenile ALS	X	X		X	X				
DAO	D-amino acid oxidase	ALS	X								
PPAR- GC1A	Peroxisome proliferator-activated receptor gamma coactivator 1-alpha	ALS	X	X							
OPTN	Optineurin	ALS, FTD		X			X	X			
CHCHD10	Coiled-coil-helix-coiled- coil-helix domain containing 10	ALS, ALS-FTD, FTD, cerebellar ataxia, myophathy	X	X		X					
NEK1	NIMA Related Kinase 1	ALS, ALS-FTD	X	X	X				X		
KIF5A	Kinesin family member 5A	ALS			X						
NEFH	Neurofilament heavy subunit	ALS			X						
TUBA4A	Tubulin Alpha 4a	ALS			X						
DCTN1	Dynactin subunit 1	ALS, ALS-FTD			X			X			
PFN1	Profilin 1	ALS			X	X					
ELP3	Elongator protein 3	ALS, ALS-FTD			X					X	
EPHA4	EPH receptor A4	ALS			X						
C9orf72	Chromosome 9 open reading frame 72	ALS, ALS-FTD, FTD				X	X	X		X	
PRPH	Peripherin	ALS			X						
CHMP2B	Charged multivesicular body protein 2B	ALS, FTD				X	X	X			
VCP	Valosin containing protein	ALS, ALS-FTD, FTD, IBM, PDB				X	X	X			
FIG4	Phosphoinositide 5-Phosphatase	ALS, PLS, CMT					X	X			
VAPB	Vesicle-associated membrane protein-associated protein B/C	ALS, PMA				X		X			
UBQLN2	Ubiquilin 2	ALS, ALS-FTD, juvenile ALS				X	X				
TBK1	TANK binding kinase 1	ALS, FTD				X	X				X
SQSTM1	Sequestosome 1	ALS, ALS-FTD, FTD, IBM, PDB				X	X				
CCNF	Cyclin F	ALS, ALS-FTD				X					
TARDBP	TAR DNA binding protein	ALS, ALS-FTD, FTD							X		
hnRNPA1	Heterogeneous nuclear ribonucleoprotein A1	ALS, ALS-FTD, FTD, IBM, PDB				X				X	
hnRN- PA2B1	Heterogeneous nuclear ribonucleoprotein A2/B1	ALS, ALS-FTD, FTD, IBM, PDB				X				X	
ALS2	Alsin	Juvenile ALS, infantile HSP						X			
SPG11	Spatacsin vescicle trafficking associated	Juvenile ALS, HSP			X	X		X	X		
SIGMAR1	Sigma non-opioid intracellular receptor 1	Juvenile ALS, dHMN					X				
C21orf2	Cilia- and flagella-associated protein 410	ALS							X		
SETX	Senataxin	Juvenile ALS, AOA2, dHMN							X	X	
FUS	Fused in sarcoma	ALS, ALS-FTD, FTD							X	X	
ATXN2	Ataxin 2	ALS, SCA2						X		X	X
ANG	Angiogenin	ALS, ALS-FTD								X	
MATR3	Matrin 3	ALS, ALS-FTD, distal myopathy								X	
EWSR1	EWS RNA binding protein 1	ALS								X	
TAF15	TATA-box binding protein associated factor 15	ALS								X	

**The table lists genes thought to be causative or risk factors for ALS sorted on the basis of their functional similarity. ALS, Amyotrophic lateral sclerosis; FTD, Frontotemporal dementia; PMA, Progressive muscular atrophy; IBM, Inclusion-body myositis; PDB, Paget disease of bone; HSP, Hereditary Spastic Paraplegia; dHMN, Distal Hereditary Motor Neuropathy; AOA2, Ataxia with oculomotor apraxia type 2; SCA2, Spinocerebellar ataxia type 2.**

Another important factor increasing the complexity of phenotype-genotype correlations in ALS is the observation of a clinical pleiotropy for ALS genes. Although some mutations associate with very specific ALS clinical profiles (e.g., patients with the Ala4Val mutation in *SOD1* usually have an aggressive form of ALS, whereas those with the homozygous Asp91Ala mutation tend to have a very slowly progressive disease with a generally ascending upper motor neuron phenotype), the majority of disease-causing genes show a high degree of phenotypic heterogeneity, with mutations in the same gene giving rise to different clinical entities, supporting a genetic basis for the observed clinical heterogeneity in ALS. A striking example of pleiotropy is due to *C9orf72* hexanucleotide repeat expansion mutation, which is clearly linked to ALS and FTD but pathogenic expansions have been also observed in a small percentage of patients affected by Alzheimer’s (<1%), Huntington’s (1–5%), and Parkinson’s diseases (1%), as well as atypical parkinsonian syndromes, such as progressive supranuclear palsy (1–8%), corticobasal degeneration (3%), and Lewy body dementia (2%) (van Blitterswijk et al., 2014b; Al-Chalabi et al., 2017; Balendra and Isaacs, 2018; Bourinaris and Houlden, 2018; Foxe et al., 2018). Another interesting example regards a newly identified ALS gene, *KIF5A.* In fact, missense mutations in the N-terminal motor domain of this gene are known to cause hereditary spastic paraplegia and Charcot–Marie–Tooth disease type 2, while ALS-associated mutations are predominantly located at the C-terminal tail domain (Brenner et al., 2018; Nicolas et al., 2018). The possible existence of a common genetic background in neurodegeneration is also supported by the observation that mutations in *ATXN2, SPAST, FIG4, SETX, DCTN1, MATR3, CHCHD10, SQSTM1, VAPB, HNRNPA1, VCP, APOE*, and *OPTN* have been reported both ALS and other multisystem disorders, including FTD, spinocerebellar ataxias, parkinsonism and schizophrenia. Among these, *APOE*, the most prevalent genetic risk factor of AD, has been also studied both as a risk factor for ALS and as a modifier of various phenotypic aspects, including age at onset, site of onset, and duration of the disease. As already found for AD, inheritance of *APOE* alleles is associated with differences in the clinical course of ALS (with a protective role of E2 allele and a deleterious role of E4 allele) suggesting a potential implication of *APOE* genotype as a biomarker to discriminate clinical efficacy in ALS clinical trials (Moulard et al., 1996; Lacomblez et al., 2002; Li et al., 2004). Another genetic determinant of ALS is the trinucleotide repeat expansion occurring in the *ATXN2* gene, with long-expanded repeats that are found to cause spinocerebellar ataxia 2 while intermediate-length polyQ expansion seems to increase the risk of developing ALS, significantly correlate to a spinal phenotype, and associate with shorter survival (Laffita-Mesa et al., 2013; van Blitterswijk et al., 2014a; Borghero et al., 2015; Chiò et al., 2015; Sproviero et al., 2017). As for mutant *C9orf72* and other pathological repeats, *ATXN2*-mediated toxicity seems to involve the creation of small toxic homopolymeric proteins, called dipeptide repeats (DPRs), through a process known as repeat-associated non-ATG-initiated (RAN) translation, leading to an impairment of ribosomal biogenesis, nucleocytoplasmic transport, RNA metabolism and protein sequestration, that can cause neurodegeneration and behavioral deficits (Barker et al., 2017; Hutten and Dormann, 2019; Hergesheimer et al., 2020). Disease-modifying therapies designed or formulated to specifically target the *ATXN2* gene, including the use of antisense oligonucleotides, are currently being studied as a promising therapeutic approach for ALS (Van Den Heuvel et al., 2014; Scoles and Pulst, 2018; Hergesheimer et al., 2020).

Besides clinical diagnosis and identification of risk variants and disease modifiers, the genomic analysis may be helpful for explaining the considerable differences in prognostic profiles of ALS patients, thus providing valuable information for designing new therapeutic strategies (Geyer et al., 2009; Tanaka et al., 2013; Su et al., 2014; Cappella et al., 2019; Chiò et al., 2020). In particular, mutations in *SOD1*, *EPHA4, KIFAP3*, and *UNC13A* seem to affect the progression of ALS disease or the survival of ALS patients (Landers et al., 2009). Loss-of-function mutations in *EPHA4* results in significantly longer survival of ALS patients and pharmacological inhibition of EPHA4 signaling has demonstrated to improve functional performance and motor neuron survival in ALS animal models (Van Hoecke et al., 2012; Rué et al., 2019). Other genetic variants associated with ALS survival include Asp91Ala, one of the most common mutations in *SOD1* that is associated with a long survival when the locus had homozygous genotype, while that of affected heterozygotes varies; and the rs12608932 located in intron 21 of the *UNC13A* gene that is associated with an increased risk and shorter survival of ALS patients (Daoud et al., 2010; Diekstra et al., 2012; Harms and Baloh, 2013; Cady et al., 2015; Gaastra et al., 2016; Yang et al., 2019).

In addition to genetic mutations, the screening of submicroscopic chromosomal changes, known as copy-number variations (CNVs), is potentially informative of genomic alterations related to disease phenotype through the modulation of the expression and function of genes. Several studies have investigated the involvement of these variants in ALS, demonstrating their involvement as risk factors, with multiple rare CNVs more important than common ones (Blauw et al., 2008, 2010; Wain et al., 2009; Uyan et al., 2013; Butchbach, 2016; Morello et al., 2018a; Vadgama et al., 2019). In particular, a large number of rare and novel ALS-specific CNV loci were identified in ALS patients, with the majority of these variants exerting a role in biochemical pathways relevant to ALS pathogenesis, including regulation of synaptic transmission and neuronal action potential, immune response and inflammation, cell adhesion, ion transport, transcriptional regulation and mRNA processing (Wain et al., 2009; Blauw et al., 2010; Morello et al., 2018a). One of the most interesting example is represented by the survival motor neuron (SMN) genes, whose copy number alterations seems to increase risk of developing SALS as well as other neurodegenerative disorders, including progressive muscular atrophy (PMA) (Blauw et al., 2012; Butchbach, 2016; Sangare et al., 2016; Morello et al., 2018a). However, other studies have not found any significant association between the deletion of either SMN1 or SMN2 in ALS, suggesting these conflicting results may be due, in part, to the existence of heterogeneous subgroups of ALS patients. The same ambiguous results are found for copy number changes affecting mitochondrial DNA (mtDNA), with some ALS patients characterized by an accumulation of deletions and other cases showing increased mtDNA copy numbers (Mawrin et al., 2004; Keeney and Bennett, 2010; Morello et al., 2018a). Other examples are heterozygous deletions of *EPHA3*, which seem to confer a protective role against the risk of developing ALS, and deletions in *NEFL* associated with a delayed disease onset and slowed disease progression (Uyan et al., 2013; Morello et al., 2018a).

Notwithstanding the increased knowledge of ALS from a genomic perspective, substantial dilemmas remain from a clinical perspective and large-scale NGS and GWAS projects are currently underway to fully unravel the underlying causes. Among these, of note is Project MinE, an international, large-scale research initiative devoted to discovering genetic causes of ALS by performing whole-genome sequencing of at least 15,000 ALS patients and 7,500 controls, resulting in an open-source genome database, in conjunction with the collection of skin samples to make patient induced pluripotent stem cell lines (iPSCs) (Van Rheenen et al., 2018; van der Spek et al., 2019). Future follow-up studies will be necessary to shed light on the biological drivers of disease and evaluate the direct effect of newly discovered genes on disease diagnosis and management, also determining if they could form candidates for novel gene therapies.

### Transcriptomic

Changes in gene expression are widespread in ALS, as revealed by a large body of work on gene expression profiling of RNA samples from peripheral cells or post-mortem nervous tissue of ALS patients and animal models. These signature patterns of gene expression have started to provide a more detailed picture of molecular events implicated in ALS pathobiology (Dangond et al., 2004; Malaspina and de Belleroche, 2004; Jiang et al., 2005; Kirby et al., 2005; Pasinelli and Brown, 2006; Wang et al., 2006; Lederer et al., 2007; Malaspina et al., 2008; de Oliveira et al., 2013; Saris et al., 2013; Raman et al., 2015; Maria D’erchia et al., 2017; Krokidis and Vlamos, 2018; Recabarren-Leiva and Alarcón, 2018; Dickson et al., 2019; Rahman et al., 2019).

**FIGURE 2:**
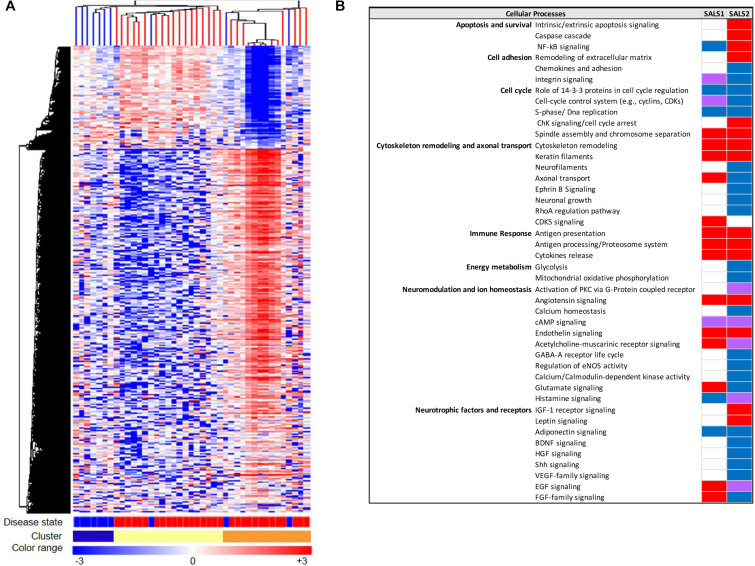
Molecular-based classification of SALS. **(A)** Unsupervised hierarchical clustering (similarity measure: Pearson centered; linkage rule: average) was used to cluster control (10 fresh-frozen motor cortex samples from non-neurological patients) and SALS patients (31 fresh-frozen motor cortex samples) on the basis of the similarity in expression profiles of the most “hypervariable genes” (9.646 genes with a standard deviation >1.5). The same clustering method clearly distinguished two SALS subgroups (SALS1 and SALS2), each associated to differentially expressed genes and pathways. In this two-dimensional presentation, each row represents a single gene and each column a motor cortex from control or SALS patients. As shown in the color bar, highly expressed genes are shown in orange, down-regulated genes in blue, no change in white. In the dendrograms shown (left and top), the length and the subdivision of the branches display the relatedness of the expression of the probes and the motor cortex (top). The disease state is marked as follows: controls patients are indicated by brown rectangles and SALS patients by red rectangles. In the cluster panel, red rectangle refers to control patients, brown to SALS1 and blue to SALS2 patients. For further details, the reader is referred to Aronica et al. (2015). **(B)** Functional pathways deregulated in clustered SALS patients. Orange boxes represent signaling pathways significantly up-regulated, blue bars down-regulated, green bars both up- and down-regulated, white bars indicate no significant change. Figure adapted from Morello and Cavallaro (2015).

The advent of systems biology and development of high-throughput technologies, including RNA sequencing and high-density microarray platforms, is enabling us not only to discover and define mechanisms of pathogenesis in ALS, but also to differentiate ALS from the “ALS mimic syndromes” and healthy controls and stratify ALS patient into subgroups, facilitating the discovery of biomarkers and new individualized treatments for patients (Cooper-Knock et al., 2012; Heath et al., 2013; Krokidis and Vlamos, 2018; Recabarren-Leiva and Alarcón, 2018; Krokidis, 2020). In this regard, our research group, in the last years, has taken important steps toward the characterization of a biological and molecular heterogeneity of ALS based on transcriptional profiles. In particular, unsupervised hierarchical clustering of genome-wide transcriptomic profiles generated from post-mortem motor cortex samples from SALS patients has led to separate healthy controls and SALS patients and identify two distinct patient groups (SALS1 and SALS2) depending on the combinations of genes and pathways that were deregulated (Aronica et al., 2015). In particular, we observed that cell death, antigen processing and presentation and regulation of chemotaxis were the most representative subgroup-specific pathways in SALS1, while deregulated genes in SALS2 were associated with axonal guidance, oxidative and proteotoxic stress (Figure 2; Aronica et al., 2015; Morello et al., 2017a,b). Our analysis also showed that some of the deregulated genes in SALS patients were previously associated with FALS, further supporting the existence of common pathological events between two disease forms. Interestingly, we found the differential expression of a substantial number of genes encoding splicing factors in the motor cortex and spinal cord of the same SALS cohort (La Cognata et al., 2020). In particular, we observed transcriptional deregulation across the tissue types and/or disease states (SALS1, SALS2, controls), with expression changes that were more pronounced for the motor cortex regions than the spinal cord and revealing a significant trend of overexpression for the SALS1 group and a decreased trend in expression for SALS2 (La Cognata et al., 2020). Despite, taken together, our results provided a powerful means for revealing etiopathogenetic mechanisms that were not emerged by considering SALS as a single pathology, it is clear that to successfully translate this knowledge to the real-world clinical contexts, the number of biomarkers should be limited. For this purpose, we next asked if the transcriptome-based classification can be reproduced by utilizing just a list of 203 genes highly associated with an increased ALS susceptibility (Morello et al., 2017a,b). Our results showed that this restricted gene panel was sufficiently representative to separate control from SALS patients, reproducing our previous classification of these patients into molecularly defined and biologically meaningful subtypes and, consequently, facilitating the identification of promising cluster-specific biomarkers. Further studies will be necessary to investigate if peripheral tissues or easily accessible biological fluids (e.g., peripheral blood monocytes, cerebrospinal fluid, or muscle) can reproduce specific molecular patterns observed in brain regions of ALS patients, allowing for an effective mechanism-based selection of patients for clinical trials of molecular-targeted therapies. Emerging molecular heterogeneity of ALS lays the foundations for developing new therapeutic strategies, targeting disease pathogenesis as a complex system rather than at the level of the single protein molecule and that may have greater relevance to distinct sets of patients. In this regard, altered biological pathways emerged from our analysis provided a good number of potential subgroup-specific biomarkers and therapeutic targets, opening the way to the implementation of genomics-based personalized medicine (Morello and Cavallaro, 2015; Morello et al., 2015, 2017c). Of note, some of these target genes exhibit expression profiles similar to those observed in animal models of ALS, thus providing a rationale to ensure their preclinical trial success (Apolloni et al., 2017; Morello et al., 2017c; Apolloni et al., 2019).

Recently, a good number of studies investigated and confirmed the existence of distinct molecular-based clusters of ALS patients, calling attention to the need for better understanding their mechanistic underpinnings and developing treatments based on specific forms of ALS (Jones et al., 2015; Tam et al., 2019; Vijayakumar et al., 2019). In particular, Tam et al. (2019) were able to stratify the transcriptomes by RNA-seq of a largely sporadic set of ALS patients’ motor cortex samples into three distinct molecular subgroups, two of which overlapped the molecular signatures observed in our ALS patient samples (Tam et al., 2019). Another study compared brain transcriptome profiles in SALS cases carrying and not carrying the *C9orf72* repeat expansion, revealing both shared and distinct transcriptome changes and pathways associated with these two subsets of ALS cases (Prudencio et al., 2015). A further interesting aspect is the possibility of separating rapid and slow ALS in earlier phases of drug development. To this regard, whole-genome expression analysis conducted by Nardo et al. (2013) in ALS animal models identified specific key genes and molecular pathways associated with fast or slow disease progression, highlighting their role as putative molecular targets for future therapeutic strategies (Prudencio et al., 2015).

The majority of the above-described studies assessed RNA samples from postmortem brain tissues. Although they provide essential elements in the pathophysiology of ALS that cannot be otherwise obtained through other approaches used in living patients, these studies reveal end-stage pathogenic mechanisms and do not clarify whether transcriptional differences that separate patient subtypes are a cause or a consequence of the disease process. In that context, the use of iPSC derived from patients suffering from ALS has provided important insights into disease pathophysiology, enabling researchers to explore molecular heterogeneity of ALS and follow the course of degeneration in the dish (Coatti et al., 2015; Bohl et al., 2016; Hedges et al., 2016; Myszczynska and Ferraiuolo, 2016; Sances et al., 2016; Csobonyeiova et al., 2017; Guo et al., 2017; Selvaraj et al., 2017; Centeno et al., 2018; Fujimori et al., 2018; Ghaffari et al., 2018; Lee et al., 2018; Halpern et al., 2019; Ziff and Patani, 2019; Chang et al., 2020; Hawrot et al., 2020). In addition, transcriptome studies on whole tissue (i.e., motor cortex and spinal cord) fail to capture dynamic changes and the complex heterogeneity of the nervous system, making it difficult to determine how gene expression changes disrupt functional interaction between motor neurons and non-neuronal cells (e.g., microglia, oligodendroglia, and astroglia) implicated in ALS pathology. Promising approaches, such as laser capture microdissection (LCM) coupled with RNA sequencing, offer a previously unavailable view of disease progression in ALS, enabling us to explore cell type-specific changes involved in the disease at a particular time point (Namboori et al., 2019; Liu et al., 2020). In a recent paper, Maniatis et al. (2019) used new RNA-seq based technologies, which they called “spatial transcriptomics,” for mapping gene expression changes occurring at different disease stages and in different regions of murine models of ALS and human postmortem spinal cords samples, providing important clues for identifying disease-associated pathways and establishing the key steps in motor neuron degeneration observed in ALS (Maniatis et al., 2019).

### Proteomics

Detection of specific protein changes in affected brain tissue samples, cell cultures or body fluids such as CSF represents an important pillar in ALS. The discovery of protein biomarkers for ALS, in fact, may aid earlier diagnosis, measure disease progression, exclude other ALS-mimicking syndromes, discriminate between subtypes of ALS that may theoretically respond to different therapeutic strategies and monitoring drug efficacy during clinical trials (Ruegsegger and Saxena, 2016; Barschke et al., 2017; Webster et al., 2017; Chipika et al., 2019; Hedl et al., 2019; Yerbury et al., 2020). It is well established that the key neuropathological hallmark of the disease is the accumulation of misfolded cytoplasmic proteins in degenerating motor neurons and their non-neuronal neighbors (Perlson et al., 2010; Baloh, 2011; Prell et al., 2013; Tang, 2014; Navone et al., 2015; Parakh and Atkin, 2016; Nguyen et al., 2019; Malik and Wiedau, 2020). One of the main protein components of these protein aggregates is TDP-43, a nuclear RNA binding protein that under stress conditions or when mutated translocates to the cytoplasm where it is hyperphosphorylated and forms insoluble ubiquitin-positive aggregates (Baloh, 2011; Weskamp and Barmada, 2018). Such aggregates are present in almost all cases of ALS, including SALS and FALS patients with pathogenic variants of *C9ORF72* (Mackenzie et al., 2007; Chew et al., 2015), as well as in other neurodegenerative disorders, including FTD, Parkinson’s and Alzheimer’s disease (Amador-Ortiz et al., 2007; Umoh et al., 2018). It is interesting to note that ALS and FTD have different forms of TDP-43 pathology, suggesting its utility for designing novel diagnostic procedures that could discriminate against these two diseases (Mackenzie and Rademakers, 2008). In addition, although controversial, several results reported that TDP-43 aggregates occur in the vast majority of *SOD1*- and *FUS*-negative FALS patients, but not in *SOD1/FUS* mutation carriers, suggesting that mutant *TDP-43* may cause ALS through specific pathways of inclusion formation that are distinct from those that underlie SALS or other FALS-associated mutations, opening the way to the development of specific therapeutic approaches that take into account these selective modifications (Farrawell et al., 2015; Jeon et al., 2019).

Due to the complex and heterogeneous nature of ALS, it is plausible that a single biomarker could not detect or differentiate between disease subgroups and/or control subjects, sustaining the importance of developing biomarker panels for specific and sensitive diagnostic tests. Recent development of high-throughput Mass Spectrometry-based proteomic (MS) technologies has allowed the simultaneous analysis of multiple proteins, allowing for the definition of comprehensive lists of possible candidate ALS biomarkers (Ekegren et al., 2008; Krüger et al., 2013; Collins et al., 2015; Hedl et al., 2019). In this regard, due to its proximity to the central motor system, the cerebrospinal fluid (CSF) may most probably reflect disease-related alterations, including changes in protein expression, post-translational modification or biochemical turnover than in other body fluids (i.e., blood or urine) (Bowser et al., 2006; Pasinetti et al., 2006; Ryberg and Bowser, 2008; von Neuhoff et al., 2012; Krüger et al., 2013; Lehmer et al., 2017). Analyses of the CSF proteome of ALS patients revealed a panel of candidate biomarkers implicated in synaptic activity, extracellular matrix, inflammatory processes, glial response, axonal damage and apoptosis (Bowser et al., 2006; Pasinetti et al., 2006; Ryberg and Bowser, 2008; von Neuhoff et al., 2012; Krüger et al., 2013; Collins et al., 2015; Collins, 2016; Lehmer et al., 2017; Benatar et al., 2018; Zubiri et al., 2018; Online et al., 2019; Zhu et al., 2019; Oeckl et al., 2020). It is interesting to note that many candidate ALS protein biomarkers show subgroup-specific differential mRNA expression in SALS patients, suggesting their utility in patient stratification and personalized medicine (Table 2). Among the most extensively studied fluid biomarkers correlating with the survival of ALS patients, higher levels of neurofilament light chain (NF-L) and the phosphorylated form of neurofilament heavy chain (pNFH) in CSF and plasma samples, as well as their accumulation in brain tissue, have been correlated to shorter life expectancy and a more rapid disease progression and have demonstrated high sensitivity and specificity for separating ALS from ALS-mimic disorders (Gaiottino et al., 2013; Lu et al., 2015; Benatar et al., 2018; De Schaepdryver et al., 2018, 2019; Verde et al., 2019). Recent works also demonstrated the diagnostic utility of CSF pNFH levels in *C9ORF72*-ALS patients, revealing higher pNFH levels in ALS or ALS/FTD patients carrying *C9ORF72* expansion compared with controls and other ALS or ALS/FTD patients (Balendra et al., 2017; Gendron et al., 2017; Floeter and Gendron, 2018). Several other proteins in CSF of ALS patients have demonstrated elevated sensitivity and specificity in distinguishing between ALS patients and neurological disease controls, including IL-10, IL-6, GM-CSF, IL-2, and IL-15 (Mitchell et al., 2009). Proteomic profiling of CSF also identified proteins with a potential prognostic value in ALS, including MIP-1α, wrCRP, HMGB, creatine kinase, granzyme B, and IL-8, whose increased levels have been correlated with more rapidly progressive disease; cystatin C protein levels were positively correlated with survival; increase in GPNMB and UCHL1 were specific for ALS patients showing a short survival time; bFGF increased in ALS patients with longer survival, whereas VGF levels correlated with progressing muscle weakness (Ranganathan et al., 2005; Barschke et al., 2017).

**TABLE 2:** Putative protein biomarkers and their differential expression in distinct SALS patient subgroups.

					Gene expression in SALS motor cortex*	
**Biomarker symbol**	**Biomarker name**	**CSF/Serum/Plasma**	**Prognostic/Diagnostic value**	**References**	**SALS1**	**SALS2**
**Neuron specific**						
MAPT	Microtubule-associated protein tau	CSF	Disease progression	(164)	↑	↓
NEFH	Neurofilament, heavy polypeptide	CSF	Diagnosis and progression	Rosengren et al., 2002; Brettschneider et al., 2006	–	↓
NEFM	Neurofilament, medium polypeptide	CSF	Diagnosis and progression	Rosengren et al., 2002	–	↓
NEFL	Neurofilament, light polypeptide	CSF	Diagnosis and progression	Rosengren et al., 2002; Zetterberg et al., 2007	–	↓
**Hormones and growth factors**						
VEGFA	Vascular endothelial growth factor A	CSF	Diagnosis and progression	Moreau et al., 2006; Pasinetti et al., 2006; Zhao et al., 2008	–	↓
GDNF	Glial cell-line derived neurotrophic factor	CSF	Diagnosis	Tanaka et al., 2006	↓	↑
IGFBP-2	Insulin-like growth factor binding protein 2	Plasma, Serum	Diagnosis and progression	Hosback et al., 2007	–	↓
IGFBP-3	Insulin-like growth factor binding protein 3	Plasma, Serum	Diagnosis and progression	Hosback et al., 2007	↑	↑
IGFBP-5	Insulin-like growth factor binding protein 5	Plasma, Serum	Diagnosis and progression	Hosback et al., 2007	↑	↓
FGF-2	Fibroblast growth factor 2	CSF, Serum	Diagnosis	Johansson et al., 2003	–	↓
HGF	Hepatocyte growth factor	CSF	Diagnosis	Tsuboi et al., 2002	–	↓
**Inflammatory system related**						
IL2	Interleukin 2	CSF	Diagnosis	Mitchell et al., 2009	–	↑
IL4	Interleukin 4	CSF, Plasma	Diagnosis and progression	Furukawa et al., 2015	–	↑
IL5	Interleukin 5 (colony-stimulating factor, eosinophil)	Plasma	Diagnosis	Lu et al., 2016	–	↑
IL6	Interleukin 6 (interferon, beta 2)	CSF, Plasma	Diagnosis and progression	Bilic et al., 2006; Mitchell et al., 2009	–	↓
IL-10	Interleukin 10	CSF, Plasma	Diagnosis and progression	Mitchell et al., 2009; Furukawa et al., 2015; Andrés-Benito et al., 2017	–	↓
IL-13	Interleukin 13	Plasma	Diagnosis and progression	Shi et al., 2007; Lu et al., 2016	–	↑
IL-15	Interleukin 15	CSF, Plasma	Diagnosis	Mitchell et al., 2009	–	↓
TNF	Tumor necrosis factor-alpha	CSF, Plasma	Diagnosis	Andrés-Benito et al., 2017	↓	–
TNFRSF1A	Tumor necrosis factor receptor superfamily, member 1A	Serum, Plasma	Diagnosis	Andrés-Benito et al., 2017	–	↓
IFNG	Interferon, gamma	CSF, Plasma	Diagnosis and progression	Guo et al., 2017	↓	↑
TGFB1	Transforming growth factor beta 1	Plasma	Disease progression	Duque et al., 2020	–	↑
GFAP	Glial fibrillary acidic protein	CSF	Diagnosis	Benninger et al., 2016	↑	–
CXCL10	Chemokine (C-X-C motif) ligand 10	CSF	Diagnosis and progression	Tateishi et al., 2010	↓	–
**Enzymes and enzyme inhibitors**						
CST3	Cystatin C	CSF	Diagnosis	Ranganathan et al., 2005	↑	–
MMP2	Matrix metallopeptidase 2 (gelatinase A, 72 kDa gelatinase, 72 kDa type IV collagenase)	CSF, Plasma	Diagnosis	Niebroj-Dobosz et al., 2010	–	↑
MMP9	Matrix metallopeptidase 9 (gelatinase B, 92 kDa gelatinase, 92 kDa type IV collagenase)	CSF, Serum, Plasma	Diagnosis	Beuche et al., 2000; Lorenzl et al., 2002	–	↓
TIMP1	TIMP metallopeptidase inhibitor 1	CSF, Serum, Plasma	Diagnosis	Lorenzl et al., 2002; Niebroj-Dobosz et al., 2010	↑	↑
SOD1	Superoxide dismutase 1, soluble	CSF, Plasma	Diagnosis	Jacobsson et al., 2001	–	↓
CHIT1	Chitinase 1 (chitotriosidase)	CSF	Diagnosis and progression	Thompson et al., 2018	–	↑
**Others**						
TARDBP	TAR DNA binding protein	CSF	Diagnosis	Majumder et al., 2018; Kasai et al., 2019	–	↓
S100B	S100 calcium binding protein B	CSF	Disease progression	Süssmuth et al., 2003	–	↓

↑, Concentration increased in SALS patients compared to controls; ↓, Concentration decreased in SALS patients compared to controls. *Aronica et al. (2015).

As for genomics studies, systems biology-oriented approaches in proteomics play a crucial role to reveal relevant biological knowledge on pathological mechanisms that trigger the onset and progression of ALS, providing a mechanistic rationale for stratification of ALS patients based on unique molecular profiles, and identification of disease biomarkers and targets for drug efficacy measurements. In this scenario, the analysis of protein-protein interaction (PPI) networks provides the possibility to group proteins that are interacting with each other’s in functional complexes and pathways, resulting critically important in helping us to comprehend complex processes, like ALS, and identify key signaling cascades, upstream regulatory components, interactome domains, and novel disease-associated protein candidates suitable for therapeutic intervention (Rao et al., 2014; Snider et al., 2015; Shurte, 2016; Mao et al., 2017; Vella et al., 2017). In this regard, an interesting example is represented by a recent study investigating modules of co-expressed genes or proteins altered in postmortem cortex samples from patients affected by ALS, FTD, ALS/FTD, and healthy disease controls. In this work, Umoh et al. (2018) identified co-expression modules (i.e., RNA binding proteins, synaptic transmission, inflammation) differing across the ALS-FTD disease spectrum that may be useful for identifying genes associated with different clinical phenotypes along the ALS-FTD disease spectrum (Umoh et al., 2018).

### Other Omics (Metabolomics, Epigenomics, miRNomics)

In addition to genomics, transcriptomics and proteomics, the exponential advances in technologies and informatics tools have stimulated an exponential growth of other areas of biomedical science (metabolomics, epigenomics, spliceomics), offering exciting new possibilities for ALS research. In this context, metabolomics, the scientific study of chemical processes involving metabolites (e.g., sugars, lipids, amino acids, organic acids), represents the downstream of systems biology that links the genome, transcriptome and proteome to patient phenotype, providing an important key tool for discovering potential markers in health or disease (Kumar et al., 2013; Blasco et al., 2016; Lanznaster et al., 2018, 2020; Germeys et al., 2019). In the last years, thanks to the development of high-throughput technologies (such as Mass Spectrometry Combined with Liquid and Gas Chromatography), metabolomics studies identified specific metabolic markers and signatures that can discriminate ALS from controls and non-ALS cases, as well as identify distinct subgroups of SALS patients, moving research toward the development of novel targeted personalized treatments (Gross et al., 2018; Lanznaster et al., 2018). In particular, Gross et al. (2018) recently identified two subgroups of SALS case fibroblasts displaying distinct metabotropic patterns that were also observed in plasma samples from the same patients, thus providing a basis for stratify SALS patients for appropriate targeted therapies (Gross et al., 2018). Other metabolite profiling-based studies revealed significantly different metabolic profiles among FALS, SALS and ALS patients carrying different mutations in disease-causing genes (i.e., *C9ORF72, SOD1, TARDBP*, and *FUS*), suggesting the existence of distinct neurodegenerative processes associated with different subtypes of ALS (Wuolikainen et al., 2012; Jääskeläinen et al., 2019; Lanznaster et al., 2020). It is interesting to note that changes in the metabolome as well as alterations in energy metabolism, such as an increase in resting energy expenditure, often precede the development of motor symptoms in ALS and correlates to disease progression. For instance, a lipid-specific metabolic abnormality is present at the pre-symptomatic stage of ALS animal models while increased serum levels of total cholesterol, LDL, LDL/HDL ratio, and triglycerides were associated with longer survival and slower disease progression in ALS patients and animal models (Dorst et al., 2011; De Aguilar, 2019; Germeys et al., 2019). However, the relationship between lipid levels and ALS is still rather controversial and poorly understood, and some follow-up observational studies of ALS did not observe any association between dyslipidemia and the incidence of ALS (Zufiría et al., 2016; De Aguilar, 2019; Zeng and Zhou, 2019). These conflicting results may be partly due to the relatively small sample sizes often employed in these observational studies and to the fact that lipid changes can be affected by a myriad of confounding factors, including genetic, nutritional, physical and pathological factors.

Analysis of metabolite profiles can be also used to identify metabolites and biochemical pathways in ALS patients that are modified before or after treatment exposure, giving rise to a new field called pharmacometabolomics (Rattray and Daouk, 2017; Blasco et al., 2018; Lanznaster et al., 2018). To this regard, an interesting example is represented by a study that analyzed changes in metabolites and lipids composition in the plasma of ALS patients enrolled in a phase III clinical trial for assessing the effects of TRO19622 (olesoxime), a compound with neuroprotective and neurodegenerative properties (Blasco et al., 2018). This study has permitted not only to identify distinct metabolic changes that can distinguish the placebo from the olexosime group but also to reveal metabolic pathways specifically altered after treatment with olesoxime and riluzole in combination in comparison to riluzole therapy alone, supporting the value of blood metabolomic profiles as biomarkers for evaluating the individual response to drug treatments and their side effects (Blasco et al., 2018).

Another layer of complexity to the understanding of complex interactions between the genome and the environment is represented by epigenetic modifications, including DNA methylation, histone post-translational modifications, ATP-dependent chromatin remodeling and RNA-dependent gene silencing (Jirtle, 2009; Dolinar et al., 2018; Douglas, 2018; Ebbert et al., 2018; Bennett et al., 2019; Calió et al., 2020; Klingl et al., 2020; Wang et al., 2020). Several lines of evidence associate epigenome modifications to ALS development, with alterations in DNA methylation and DNA-(cytosine-5)- methyltransferase (DNMT) enzyme activity, as well as alterations to the balance between histone acetylation and deacetylation observed in blood and post-mortem neural tissue from patients with ALS and in different experimental models (Paez-Colasante et al., 2015; Dolinar et al., 2018; Bennett et al., 2019). Of note, variations in epigenetic marks and modifier enzymes, and alterations in the methylation status of some ALS-related genes promoters were also determined, including hypomethylation of *OPTN*, hypermethylation of *C9orf72* expansion CpG islands in the blood of FTD/ALS patients, whereas mutant *SOD1, FUS* and *TDP43* contribute to global epigenome alteration by inducing alterations in histone post-translational modifications and DNA methylation (Masala et al., 2018). While several high-density microarrays or sequencing-based epigenomic technologies are available, particular attention should be paid to EpiSwitch^TM^, a high-resolution platform, recently developed by Oxford BioDynamics, for analyzing structural-functional epigenetic changes in genomic architecture associated with pathological phenotypes called “chromosome conformation signatures.” Using this innovative technological platform, Salter and colleagues performed a comparative interrogation of the genomic architecture from healthy and ALS-patient blood samples revealing unique chromosomal conformation signatures with the ability to discern between diseased subjects and healthy controls, predict faster versus slower progressing patients at baseline and stratify responsive and non-responsive patients, representing a crucial step toward personalized medicine in ALS (Poesen, 2018; Salter et al., 2018).

MicroRNAs (miRNAs), small non-coding molecules of about 20–22 nucleotides, represent an additional layer of epigenetic regulation that, thanks to their capability to be highly stable in human body fluids, are considered promising biomarkers for neurodegenerative diseases, including ALS (Ricci et al., 2018; Sharma and Lu, 2018). Over the last few years, several whole-genome miRNA profiling studies have identified a panel of a dozen miRNAs that can distinguish ALS from controls with high accuracy in blood cells, serum and CSF, and may be altered in pre-symptomatic ALS mutation carriers even years before the estimated disease onset, representing potentially useful biomarkers of early-stage ALS in coming years (Figueroa-Romero et al., 2016; Rizzuti et al., 2018; Joilin et al., 2019). Despite the heterogeneous nature of ALS may prevent a significant correlation of miRNA levels with clinical disease parameters, down-regulation of two miRNAs, miR-1234-3p and miR-1825, not only is specific for ALS, at least when compared with cohorts of Alzheimer’s and Huntington’s disease, but also significantly correlated with disease characteristics like age of onset, disease severity and duration (Freischmidt et al., 2015; Takahashi et al., 2015). In particular, while the downregulation of miR-1825 is a general early feature in both FALS and SALS, miR-1234-3p is significantly downregulated only in SALS patients. Of note, a large proportion of SALS patients showed miRNA signatures resembling those of FALS patients and mutation carriers, suggesting alteration of common pathways and a high contribution of genetic factors also in SALS (Freischmidt et al., 2015; Takahashi et al., 2015). Other examples include down-regulation of miR-206, a specific modulator of skeletal muscle growth involved in nerve regeneration after injury, which accelerates disease progression in ALS mice, whereas up-regulation of miR-208B and miR-499 is found in the skeletal muscles of patients with slower disease progression, suggesting the potential utility of these microRNAs as promising candidate biomarkers and targets for this motor neuron disease (Toivonen et al., 2014; Ma et al., 2015; Di Pietro et al., 2017; De Luna et al., 2020).

## From Single Level to Multi-Omics Integrative Analyses: Toward Precision Medicine in ALS

As detailed in the previous paragraphs, omics technologies have been used to identify and/or provide functional supporting information for deciphering important players and pathways involved in ALS pathogenesis and identifying a panel of candidate therapeutic targets and biomarkers that will assist in the rapid diagnosis and prognosis assessment of the disease, and in the stratification of patients into different subgroups for specific targeted therapies. However, if considered individually, these technologies are insufficient to clarify the intricate disease mechanisms implicated in ALS. Taking a holistic molecular approach, based on the integration of multiple types of omics data with existing biological knowledge, has the potential role in improving the knowledge of the molecular basis underlying complex and heterogeneous diseases, establishing different molecular subtypes and patient stratification, thus providing a rational foundation for designing new studies to identify novel targets and clinical trials (Figure 1; Mitropoulos et al., 2018; Yu and Zeng, 2018; Mirza et al., 2019; Nguyen and Wang, 2020). Numerous studies have demonstrated the utility of whole- and multi-omics strategies for deciphering the molecular landscape of neurodegenerative diseases, including ALS, providing a feasible opportunity to develop an efficient and effective personalized diagnostics and patient-guided therapies (Bu et al., 2016; Santiago et al., 2017; Castrillo et al., 2018; Hampel et al., 2018a,b; Mitropoulos et al., 2018; Olivier et al., 2019; Vijayakumar et al., 2019; Lam et al., 2020).

An interesting example of applying integrated omics approaches to define an individual’s molecular profile useful for the development and application of personalized medicine in ALS, is represented by recent studies carried out by our research groups. As previously described, transcriptional profiling of post-mortem motor cortex samples from SALS patients has allowed to differentiate two distinct patient subgroups characterized by different deregulated genes and pathways (Aronica et al., 2015; Morello and Cavallaro, 2015; Morello et al., 2017a,b,c). To investigate whether these transcriptional alterations may be related to genomic DNA alterations, and thus represent potential markers for a molecular-based stratification of SALS patients, we integrated gene expression profiling with the analysis of genomic structural aberrations occurring in the motor cortex of the same set of SALS samples (Morello et al., 2019). This comprehensive molecular characterization at the genomic and transcriptomic levels revealed subtype-specific genomic alterations positively correlating with transcriptional signature profiles, further confirming the existence of molecular and functional heterogeneity in SALS and suggesting that genomic and transcriptomic events complement each other in driving disease pathogenesis (Morello et al., 2019). Beyond refining ALS molecular architecture, our results also pinpointed candidate driver genes potentially useful as therapeutic targets and biomarkers for genomic-based patient stratification and individualized treatment (Morello and Cavallaro, 2015; Morello et al., 2017c, 2019; Maugeri et al., 2019). Among these, numerous genes involved in histamine receptors, metabolism, transport, secretion and signal transduction, were differentially expressed in the motor cortex as well as in the spinal cord of two molecular-based subgroups of SALS patients and, of note, some of these genes are located within genomic regions disrupted by DNA copy number occurring in SALS patients (Apolloni et al., 2019). By integrating our data with the known pathogenic variants of ALS-related gene reported in the ALSOD database, we identified a good number of coding variants in these genes, supporting the hypothesis that histamine-related genes might represent candidate biomarkers and targets for patient-oriented ALS care (Apolloni et al., 2019). In this regard, pharmacological modulation of the histamine-related pathway has already proved broad efficacy in ameliorating ALS features, improving motor performance and survival in ALS mice and increasing motor neurons survival *in vivo* and *in vitro* ALS models (Apolloni et al., 2017, 2019).

## Conclusion

In the past decade, advanced omics technologies have fostered our understanding of the complex molecular architecture of ALS, contributing in part to explain its clinical heterogeneity, and providing a basis for a molecular taxonomy that may radically change our medical approach to ALS. The identification of relevant classifiers and subgroup-specific diagnostic, prognostic and predictive biomarkers is in fact urgently needed for accelerating the development of effective and personalized treatment approaches in ALS. In this review, we discuss the most significant contributions of omics approaches in unraveling the biological complexity of ALS, highlight how holistic systems biology approaches and multi-omics data integration are ideal to provide a comprehensive characterization of patient-specific molecular signatures that could potentially guide therapeutic decisions. We strongly believe that the future research in ALS, as well as in other neurodegenerative diseases, calls a multidisciplinary holistic approach, integrating multi-layer omics data with multimodal neuroimaging and clinical data. This approach will provide a clear understanding of disease prognosis and progression and accelerate development of innovative, effective and personalized strategies for ALS.

## Author Contributions

GM wrote the manuscript. SS, VD’A, and FC participated in revising the manuscript. SC conceived, directed, and supervised the project. All authors contributed to the article and approved the submitted version.

## Conflict of Interest

The authors declare that the research was conducted in the absence of any commercial or financial relationships that could be construed as a potential conflict of interest.

## Footnotes

1 https://alsod.ac.uk

## Abbreviations

ALS: amyotrophic lateral sclerosis
MNs: motor neurons
FDA: Food and Drug Administration
UMN: upper motor neuron
LMN: lower motor neuron
FTD: frontotemporal dementia
FALS: familial ALS
SALS: sporadic ALS
SNPs: single nucleotide polymorphisms
SOD1: Superoxide dismutase 1 [Cu-Zn]
C9orf72: chromosome 9 open reading frame 72
FUS: Fused in Sarcoma RNA binding protein
TDP-43/TARDBP: TAR DNA binding protein
GWAS: genome-wide association studies
WGS: whole-genome sequencing
WES: whole-exome sequencing
KIF5A: kinesin family member 5A
ATXN2: ataxin 2
SPAST: spastin
FIG4: FIG4 phosphoinositide 5-phosphatase
SETX: senataxin
DCTN1: dynactin subunit 1
MATR3: matrin 3
CHCHD10: coiled-coil-helix-coiled-coil-helix domain containing 10
SQSTM1: sequestosome 1
VAPB: VAMP associated protein B and C
HNRNPA1: heterogeneous nuclear ribonucleoprotein A1
VCP: valosin containing protein
OPTN: optineurin
EPHA4: Ephrin type-A receptor 4
KIFAP3: Kinesin Associated Protein 3
UNC13A: Unc-13 Homolog A
CNVs: copy-number variations
SMN: survival motor neuron
PMA: progressive muscular atrophy
mtDNA: mitochondrial DNA
EPHA3: Ephrin type-A receptor 3
iPSC: induced pluripotent stem cells
LCM: laser capture microdissection
MS: Mass Spectrometry
CSF: cerebrospinal fluid
NF-L: neurofilament light chain
pNFH: phosphorylated neurofilament heavy chain
IL-10: interleukin 10
IL-6: interleukin 6
IL-2: interleukin 2
IL-15: interleukin 15
IL-8: interleukin 8
GM-CSF: Granulocyte-Macrophage Colony-Stimulating Factor
MIP-1 α: Macrophage Inflammatory Proteins 1-alpha
wrCRP: wide-range C-reactive protein
HMGB: High Mobility Group Box 1
GPNMB: glycoprotein NMB
UCHL1: ubiquitin C-terminal hydrolase L1
bFGF: basic fibroblast growth factor
VGF: Nerve Growth Factor Inducible
PPI: protein-protein interaction
LDL: low-density lipoprotein
HDL: high- density lipoprotein
DNMT: DNA-(cytosine-5)-methyltransferase
miRNA: MicroRNA.
